# Cost‐effectiveness of leveraging long‐acting injectable cabotegravir to expand PrEP coverage among MSM in two contrasting North American cities

**DOI:** 10.1002/jia2.70061

**Published:** 2026-01-19

**Authors:** Jesse A. Heitner, Sarah E. Stansfield, Kate M. Mitchell, Carla M. Doyle, Rachael M. Milwid, Mia Moore, Deborah J. Donnell, Yiqing Xia, Mathieu Maheu‐Giroux, Ruanne V. Barnabas, Marie‐Claude Boily, Dobromir T. Dimitrov

**Affiliations:** ^1^ Division of Infectious Diseases Massachusetts General Hospital Boston Massachusetts USA; ^2^ Fred Hutchinson Cancer Center Seattle Washington USA; ^3^ MRC Centre for Global Infectious Disease Analysis School of Public Health, Imperial College London London UK; ^4^ HIV Prevention Trials Network Modelling Centre, Imperial College London London UK; ^5^ Department of Nursing and Community Health Glasgow Caledonian University London London UK; ^6^ Department of Epidemiology and Biostatistics, School of Population and Global Health McGill University Montréal Québec Canada; ^7^ Department of Medicine Harvard Medical School Boston Massachusetts USA; ^8^ University of Washington Seattle Washington USA

**Keywords:** cabotegravir, cost‐effectiveness analysis, emtricitabine tenofovir disoproxil fumarate drug combination, HIV prevention, men who have sex with men, PrEP

## Abstract

**Introduction:**

Long‐acting injectable cabotegravir (CAB‐LA) is superior to daily oral tenofovir disoproxil fumarate/emtricitabine (TDF/FTC) for HIV pre‐exposure prophylaxis (PrEP) and could expand PrEP usage. Given price differentials between CAB‐LA and TDF/FTC, evaluating the cost‐effectiveness of potential PrEP coverage scenarios is warranted.

**Methods:**

We simulated PrEP coverage expansion among men who have sex with men (MSM) via introducing CAB‐LA using two age‐ and risk‐stratified HIV transmission models separately calibrated to local data from a high‐incidence (Atlanta, USA) and a low‐incidence (Montréal, Canada) North American setting. PrEP coverage of HIV‐negative MSM was simulated to increase from 6% to 15%, 30%, 40% or 50% (Montréal) or from 29% to 40% or 50% (Atlanta), within 5 or 10 years, with 0%, 15%, 30%, 50% or 100% of current TDF/FTC users switching to CAB‐LA. Costing took a healthcare payer perspective and included PrEP pharmaceuticals, PrEP programmatic costs and HIV‐related care. Atlanta scenarios considered oral PrEP acquired at average recent market prices (primary analysis), and both settings modelled universal acquisition at the lowest available generic price (LAGP). Simulations were compared to baseline projections without CAB‐LA‐based expansions over 20 years, with costs and disability‐adjusted life years (DALYs) discounted 3% annually. Incremental cost‐effectiveness ratios (ICERs) of expansions were assessed against a $100,000 per DALY averted threshold.

**Results:**

In Atlanta, scenario median ICERs at recent prices ranged from $141,600 (90% CI $60,100−$256,000) to $203,800 ($99,300−$359,200) per DALY averted. All uncertainty intervals covered $100,000. Under universal LAGP TDF‐FTC, median ICERs ranged from $255,800 ($112,900−$452,30) to $370,700 ($172,200−$669,100). The strongest expansion scenarios were expected to remain cost‐effective until approximately $2800/dose, or approximately $1350 with universal LAGP TDF/FTC. In Montréal, scenarios had median ICERs from $920,000 to $2,540,000, excluding dominated runs.

**Conclusions:**

In a high‐incidence Atlanta MSM population, CAB‐LA‐based PrEP expansions are not projected to be cost‐effective, though a minority of simulations achieved cost‐effectiveness. However, lower prices could achieve cost‐effectiveness. In a low‐incidence Montréal MSM population, broad expansions are not expected to be cost‐effective at modelled prices. Prioritizing CAB‐LA to Montréal MSM facing access, adherence or persistence barriers to oral PrEP warrants a cost‐effectiveness assessment.

## 1 INTRODUCTION

Two randomized trials (HPTN 083 and HPTN 084) demonstrated that long‐acting cabotegravir (CAB‐LA) injected every 8 weeks is superior to daily oral tenofovir disoproxil fumarate/emtricitabine (TDF/FTC) for pre‐exposure prophylaxis (PrEP) to prevent HIV acquisition in cisgender men who have sex with men (MSM), transgender women (TGW) [1] and cisgender women [2]. CAB‐LA has since been approved for PrEP in the United States (in 2021) and Canada (in 2024) [3, 4].

PrEP is underutilized in both Canada [5, 6] and the United States [7, 8]. At the end of 2021, only 34% of cisgender MSM in the United States with a PrEP indication were prescribed it [9]. Preference studies outside of trial settings indicate large proportions of US MSM (25−67%) self‐report preferring an injectable over oral PrEP [10, 11, 12, 13]. Trial settings suggest even stronger preferences. Of participants within the open‐label extension of HPTN 083, 96% chose CAB‐LA [14]. Similarly, in the phase IIa ÉCLAIR treatment trial, of those receiving consecutive injections, 74% favoured CAB‐LA over oral cabotegravir [15]. There is optimism that adding a preferable and more effective PrEP option could increase coverage [16].

CAB‐LA's introductory US price was much higher than the available generic oral PrEP. At $3700 per 8‐week injection ($24,050 annually) [17], it was comparable to prices for branded oral TDF/FTC ($21,600 annually) and the similar, non‐inferior branded emtricitabine/tenofovir alafenamide (TAF/FTC; $22,800 annually) [18, 19]. However, generic oral TDF/FTC entered the US market in late 2020, and within two quarters, its price dropped to approximately $360 annually [20]. Thus, while clinically effective, the cost‐effectiveness of CAB‐LA is in question.

Two models published since the HPTN 083 results announcement have investigated CAB‐LA cost‐effectiveness for MSM and TGW in the United States with differing methodologies and strikingly different conclusions. Neilan and colleagues found that for a cost‐effectiveness threshold of $100,000 per QALY, CAB‐LA would need to be priced at < $4100 annually [21]. Under the same threshold, Brogan and colleagues reported that at current prices, starting a PrEP user on CAB‐LA, with subsequent medication switching allowed, achieved an incremental cost‐effectiveness ratio (ICER) of $46,000 per QALY gained [22]. To our knowledge, these are the only post‐trial, peer‐reviewed published estimates available for North America. Their disparity in conclusions could give rise to confusion for large‐scale healthcare financers and regulators. Moreover, both assess dichotomized options generally formulated as choosing CAB‐LA initiation versus oral PrEP initiation, with neither assessing the population‐average cost effectiveness of CAB‐LA introduction for a population that is likely to both expand and to make a mixture of initiation choices.

Recent modelling analysis estimated that increasing overall PrEP coverage by offering CAB‐LA to MSM in Atlanta and Montréal could avert a large proportion of new HIV acquisitions over 2022−2042 [23]. We build upon these prior results to investigate whether the health benefits they project are cost‐effective at the population scale. Their sharply contrasting baseline epidemics allow insight into how cost‐effectiveness may vary with incidence. Atlanta is one of the US cities with the highest HIV prevalence among MSM. In contrast, Montréal, Canada, is a setting of comparatively low HIV incidence among MSM, and was the first Canadian Fast‐Track City, pledging to end the HIV/AIDS epidemic by 2030. Both cities have been past subjects of HIV transmission dynamic modelling [6, 16, 24, 25, 26].

## 2 METHODS

### 2.1 Health outcomes estimation

Different age‐ and risk‐stratified HIV transmission models (deterministic compartmental in Atlanta; stochastic individual‐based model in Montréal) were parameterized and calibrated to local data. Baseline simulations assumed a continuance of 2021 trends, including oral PrEP usage trends, without the introduction of CAB‐LA. Given that TDF/FTC and TAF/FTC have not demonstrated statistically differing effectiveness [27], for simplicity, oral PrEP health outcomes were not modelled separately by oral option. Multiple alternative scenarios were then simulated, incorporating the introduction of CAB‐LA. PrEP coverage of MSM not living with HIV was simulated in Montréal to increase from baseline coverage of 6% [26] to 15%, 30%, 40% or 50%. In Atlanta, where oral PrEP coverage among MSM was already 29% [16], expansions to 40% or 50% were simulated. Expanded coverage above baseline projected trends was achieved by recruiting PrEP naïve MSM to CAB‐LA. Existing users and users who would have initiated oral PrEP without CAB‐LA introduction were simulated to switch to CAB‐LA in varying proportions (i.e. 0%, 15%, 30%, 50% or 100%), with existing 2022 users and those newly recruited by 2042 choosing CAB‐LA over oral PrEP in the same proportions. Coverage targets were achieved in either 5 or 10 years. See Figure S1 for a schematic example. Scenario uncertainty was assessed by simulating at least 100 replicates, each with individually calibrated parameter sets. Detailed procedures have been reported previously [23].

Here, we translate the previously reported epidemiological outcomes into the scenarios that took a prioritized coverage approach into their resulting disability‐adjusted life years (DALYs), and cost each scenario to calculate its cost‐effectiveness. In Atlanta, exposure risk groups were dichotomized as meeting US PrEP indication guidelines or not, and in Montréal, as having a high (≥11), medium (6−10) or low (≤5) number of anal sex partners annually.

DALYs calculations used standard disability weights [28], with HIV staging mapped to simulated CD4 counts in order to calculate years lived with disability (YLD) (see Table S1). Years of life lost (YLL) were based on local life tables for men [29, 30], and were considered to be incurred inclusive of the entire life expectancy during the year of death. All YLD and YLL were discounted at 3% annually in primary analyses. For Montréal, a secondary sensitivity analysis was also performed, which utilized undiscounted DALYs.

### 2.2 Cost estimation

Costing took a payer perspective. This included the annual pharmaceutical costs of either PrEP option or related programmatic PrEP costs, including lab testing as prescribed in the open‐label phase of the HPTN 083 trial protocol [31]. Additionally, we included annual incremental patient care costs per person living with HIV, including annual antiretroviral treatment (ART), laboratory, inpatient, outpatient and emergency department costs. We did not account for start‐up programmatic costs for CAB‐LA rollout, such as provider training, nor outreach costs to potential users to achieve modelled coverage levels. These costs may comprise an appreciable percentage of the total costs of achieving desired coverage levels, but as discussed in the limitations, they are difficult to predict precisely. Indirect social costs were not considered. All costs were assessed in 2021 USD, with Canadian dollars (CAD) converted to US dollars (USD) at 0.77 USD/CAD. Costs were discounted at 3% annually. Details by setting are presented below.

#### 2.2.1 Atlanta

##### Pharmaceutical costs

The initial wholesale price of CAB‐LA in the United States was $3700/dose [17]. If delivered every 8 weeks as per trial protocols [32], the average annual cost per established user is $24,050. We take this as the per‐protocol cost, though some analyses have assumed bi‐monthly injections amounting to $22,200 annually, and notably matches the midpoint of branded TDF/FTC and TAF/FTC annual wholesale prices. Manufacturer rebates may reduce the effective pharmaceutical costs to purchasers able to negotiate them. We present a sensitivity analysis varying the purchaser price down to $50/dose, an approximately 99% price reduction encompassing the plausible range of negotiable prices. Notably, the Clinton Health Access Initiative has estimated CAB‐LA manufacturing costs to be $2.00−$3.50/dose, depending on scale [33], representing a lower bound for future generic competition.

We take two approaches to costing oral PrEP pharmaceuticals for Atlanta: (1) utilizing recent US national estimated average prices ($16,380/year [34]), which reflects a mixture of branded TDF/FTC, branded TAF/FTC and available generic prices weighted by 2021 prescribing volumes, and (2) utilizing the lowest available generic price (LAGP) for all prescriptions (generic price, $360/year [20]), as the limit of the most economically efficient opportunity cost comparison.

##### Programmatic PrEP delivery costs

Both oral PrEP and CAB‐LA programmatic costs ($653/year and $1003/year, respectively) assume adherence to the HPTN 083 trial protocols. The clinical unit costs are taken from Neilan and colleagues, based on the HPTN 083 trial [21], with office visits costing $104, plus an additional $16 injection fee in the case of CAB‐LA. Laboratory costs reflect applicable Centers for Medicare & Medicaid Services reimbursement rates for trial protocol assays. These included an HIV‐1 antibody test at each visit, costing $9.09, plus additional laboratory monitoring totalling $163 annually for both PrEP options (see Table S2).

##### Incremental HIV care costs

Several estimates exist for annual and lifetime costs of healthcare in the United States for people living with HIV [35, 36, 37, 38]. We derive our average annual estimate based on Cohen et al. [36], which is noteworthy for (1) estimating the *incremental* healthcare costs incurred by people living with HIV above matched controls, (2) utilizing real‐world adjudicated claims data, and (3) being the most recent comprehensive estimate. These estimates were inflated to 2021 USD based on US medical inflation rates [39]. See Table S4 for details.

#### 2.2.2 Montréal

Costing methodology for Montréal follows that of Ouellet et al. [40], which modelled cost‐effectiveness of “on‐demand” PrEP for MSM in Montréal. On‐demand costs were scaled up to provide for daily coverage, and all prices for PrEP provision and HIV‐related care were updated to 2021 reimbursement rates from their respective sources [41, 42, 43]. Where multiple potentially relevant costs were reported, the lowest available cost was assumed.

##### Pharmaceutical costs

The price of generic TDF/FTC published by Quebec's health insurance board (RAMQ) in 2021 was 7.30 CAD/pill [41], or 2669 CAD/year (2055 USD/year).

CAB‐LA was approved for HIV PrEP by Health Canada in May 2024. In August 2024, the Canadian Agency for Drugs and Technologies in Health (CADTH) recommended its reimbursement under the sponsor‐submitted price of 1710 CAD per dose, or 10,260 CAD (7900 USD) annually, without an oral lead in [4]. However, the final recommendation was to “Reimburse with conditions,” which included price and economic feasibility considerations. Therefore, we present a main estimate based on the sponsor‐submitted price, and in Supplementary Analyses, present ICERS on a range of potential prices (see Table S7).

##### Programmatic PrEP delivery costs

We adapted the ingredients‐based approach of Ouellet et al. [40] to the visit frequencies and laboratory monitoring protocols of the open‐label phase of HPTN 083. We drew costs from the same reimbursement schedules but utilized updated lists. Programmatic costs are detailed in Table S3, and totalled CAD 807 for oral PrEP and CAD $1184 for CAB‐LA.

##### HIV care costs

Ouellet et al. [40] reported annual HIV care costs comprised of outpatient, inpatient, emergency department, psychosocial and antiretroviral costs, with medical resource utilization rates for people living with HIV. Our costs were derived from the same sources based on updated price and reimbursement lists, except for inpatient care, which was estimated by CD4 category based on work by Krentz and colleagues [44]. See Table S5 for details.

### 2.3 Scenario analysis and comparison

The ICER of each scenario replicate took the change in total payer costs from the baseline and divided by the DALYs averted from baseline. Lower ICERs indicate reduced cost per unit of health. Estimates should be interpreted as the cost‐effectiveness of the entire scenario (including CAB‐LA and oral PrEP components) over baseline trends with only oral PrEP. The distribution of each scenario's ICER is the distribution of the ICERs of its ≥100 replicates. In Atlanta, multi‐factorial ANOVA and multi‐variable regression investigated the association of scenario ICERs with PrEP coverage, switching proportions and years to achievement, with all replicates contributing ICERs. In Montréal, despite expanded PrEP coverage and CAB‐LA's greater protective effect over oral TDF‐FTC, some replicates stochastically generated increases in HIV acquisitions and/or associated DALYs. Such outlier replicates are removed from our primary reported findings, but the result tables contain additional columns that include them. Further, in Montréal, heteroskedastic results necessitated the use of non‐parametric Wilcoxon rank‐sum tests to investigate the same associations. Analyses were conducted utilizing STATA/SE 14.2.

## 3 RESULTS

### 3.1 Atlanta

**Figure 1:**
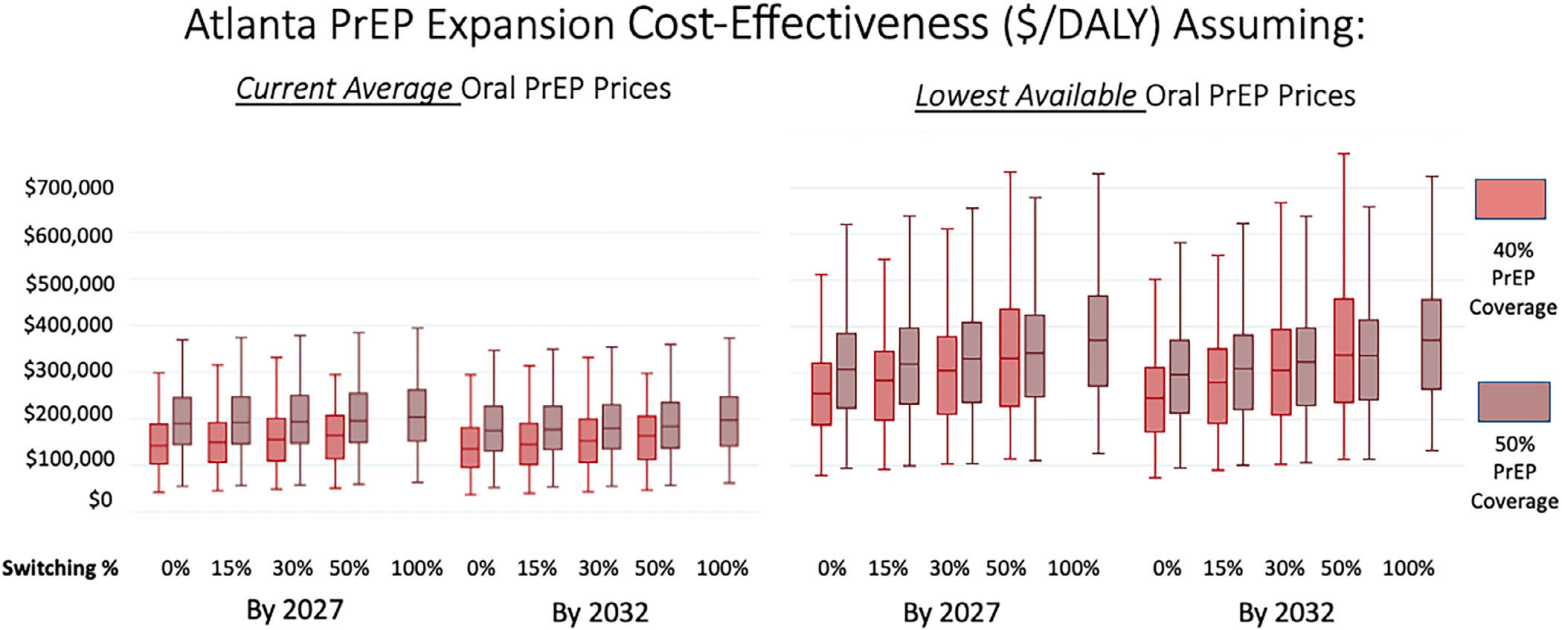
Incremental cost‐effectiveness ratios of expanding pre‐exposure prophylaxis (PrEP) coverage with long‐acting injectable cabotegravir (CAB‐LA) and exposure risk prioritization among men who have sex with men (MSM) in Atlanta (USA). *Note*: Median with inter‐quartile (boxes) range and adjacent values (whiskers). Abbreviations: CAB‐LA, long‐acting cabotegravir; DALY, disability‐adjusted life year; MSM, men who have sex with men; PrEP, pre‐exposure prophylaxis.

No Atlanta expansion scenarios resulted in median predicted ICERS under $100,000/DALY averted; however, those utilizing recent average oral PrEP prices all had uncertainty intervals that covered this threshold (Figure 1 and Table 1).

**Table 1:** Cost‐effectiveness of scenarios expanding PrEP coverage for MSM with CAB‐LA and exposure‐risk prioritization in Atlanta, 2021 USD

			Current average oral PrEP prices			Lowest available oral PrEP prices		
Coverage %	Year achieved	Switching %	5‐%ile	Median ICER	95‐%ile	5‐%ile	Median ICER	95‐%ile
40%	2027	0	*$60,129*	**$141,604**	*$255,970*	*$112,862*	**$255,772**	*$452,250*
		15	*$65,115*	**$149,012**	*$268,027*	*$123,086*	**$284,552**	*$535,716*
		30	*$65,384*	**$154,732**	*$279,172*	*$127,413*	**$304,981**	*$602,071*
		50	*$67,872*	**$163,886**	*$294,039*	*$136,012*	**$331,903**	*$684,464*
	2032	0	*$55,826*	**$134,558**	*$249,464*	*$103,966*	**$245,682**	*$443,334*
		15	*$58,648*	**$144,328**	*$263,678*	*$114,260*	**$279,792**	*$548,629*
		30	*$60,254*	**$152,178**	*$277,408*	*$123,288*	**$305,884**	*$627,510*
		50	*$64,075*	**$162,905**	*$291,631*	*$136,731*	**$338,705**	*$727,224*
50%	2027	0	*$88,919*	**$189,808**	*$336,056*	*$144,880*	**$307,666**	*$534,747*
		15	*$90,053*	**$191,628**	*$340,696*	*$150,737*	**$319,369**	*$553,556*
		30	*$91,777*	**$193,350**	*$346,064*	*$156,528*	**$330,310**	*$564,699*
		50	*$94,047*	**$195,608**	*$349,386*	*$164,087*	**$342,925**	*$589,630*
		100	*$99,344*	**$203,647**	*$359,226*	*$182,784*	**$370,551**	*$660,560*
	2032	0	*$83,121*	**$174,271**	*$310,469*	*$135,176*	**$296,490**	*$530,111*
		15	*$84,692*	**$176,903**	*$316,161*	*$140,936*	**$309,783**	*$542,332*
		30	*$86,247*	**$179,379**	*$321,782*	*$147,750*	**$323,690**	*$563,719*
		50	*$88,207*	**$183,400**	*$326,512*	*$156,567*	**$337,485**	*$595,105*
		100	*$93,515*	**$196,932**	*$345,465*	*$172,056*	**$370,742**	*$669,058*

Abbreviations: CAB‐LA, long‐acting cabotegravir; DALY, disability‐adjusted life year; ICER, incremental cost‐effectiveness ratio; MSM, men who have sex with men; PrEP, pre‐exposure prophylaxis. Median estimates are bolded; simulation‐bsed confidence bounds are italicized.

With only one weak exception, median ICERs of expansion scenarios with 50% PrEP coverage were higher than those with 40% PrEP coverage and equivalent switching and timelines. Scenarios with higher switching percentages had higher median ICERs, holding coverage and timelines equivalent. Multiple regression and multi‐factorial ANOVA indicated statistical significance for both trends (each with *p* < 0.001). Expansions achieved by 2032 typically had lower ICERs than comparable scenarios achieved by 2027, but this was not statistically significant (*p* = 0.089). ICERS determined utilizing oral PrEP's LAGP always had higher ICERs than those calculated using recent average PrEP prices (*p* < 0.001).

**Figure 2:**
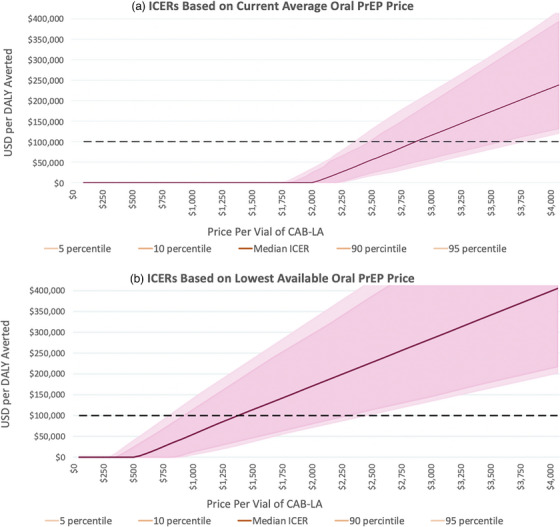
Cost‐effectiveness of a strong PrEP expansion (50% coverage by 2027 with 100% switching) at potential post‐rebate effective prices in Atlanta (USA). *Note*: Median, 80% and 90% credible intervals of USD per DALY averted. Panel A uses current average oral PrEP price landscape in ICER calculations, while panel B uses the lowest available oral PrEP price in ICER calculations. Abbreviations: CAB‐LA, long‐acting cabotegravir; DALY, disability‐adjusted life year; ICER, incremental cost‐effectiveness ratio; PrEP, pre‐exposure prophylaxis.

To investigate the possible effects of manufacturer rebates or other potential price changes, we analysed the cost‐effectiveness of the strongest modelled PrEP expansion (achieving 50% PrEP coverage by 2027 with a switching proportion of 100%) for per‐dose prices down to $50 and up to $4000 (Figure 2). Under recent average oral PrEP prices, a strong CAB‐LA based‐PrEP expansion among MSM in Atlanta would be expected to be cost‐effective up to approximately $2800 per dose and cost‐saving at prices up to $1950 (Figure 2A). In contrast, assuming universal usage of LAGP oral PrEP, CAB‐LA is confidently expected to be cost‐saving only up to $500 per dose, and retains a median projection of cost‐effectiveness up to approximately $1350/dose (Figure 2B).

#### 3.1.1 Montréal

**Figure 3:**
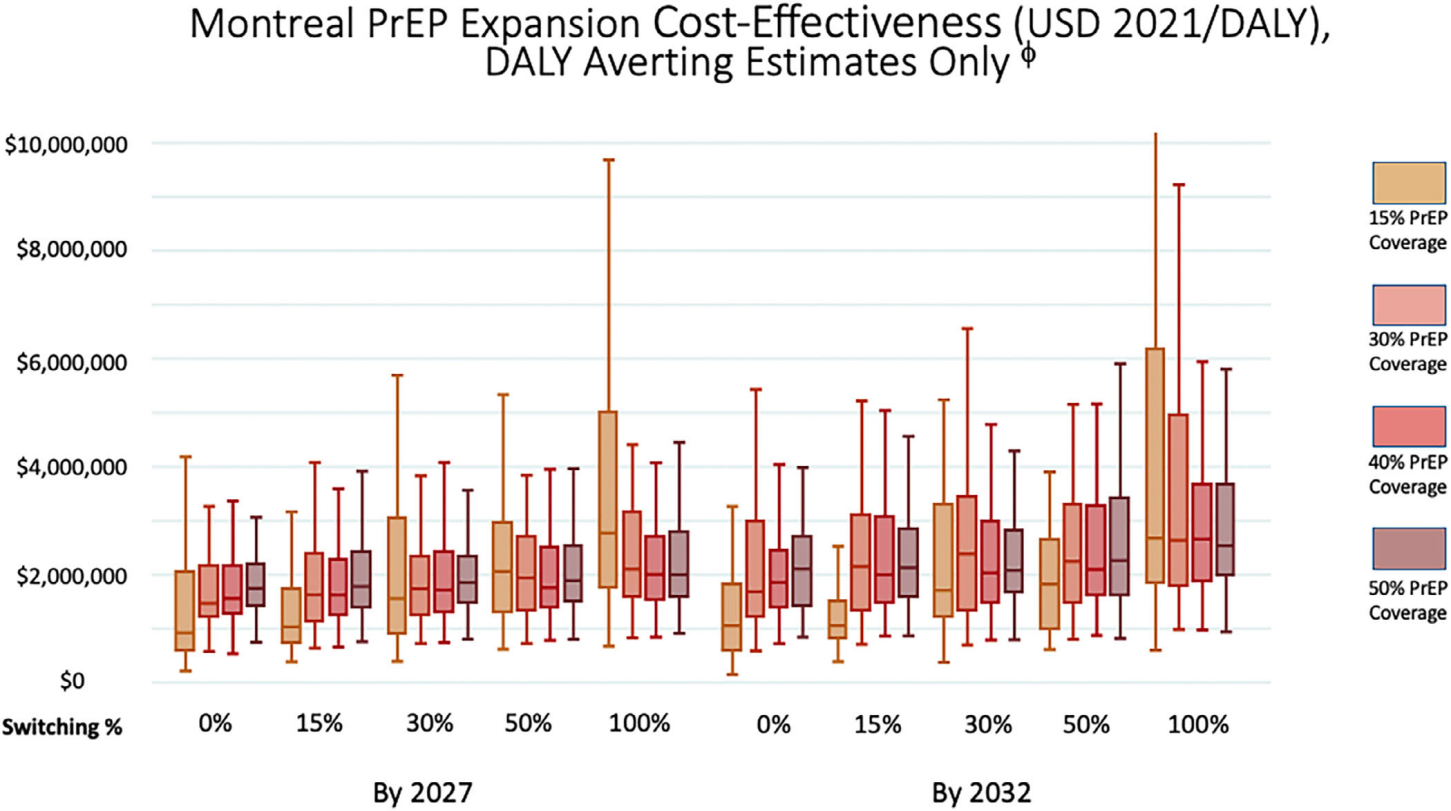
Incremental cost‐effectiveness ratios of expanding pre‐exposure prophylaxis (PrEP) coverage with long‐acting injectable cabotegravir (CAB‐LA) and exposure risk prioritization among men who have sex with men (MSM) in Montreal (Canada), with DALY increasing estimates excluded. *Note*: Median with inter‐quartile (boxes) range and adjacent values (whiskers). ϕ Included simulations by scenario (*N* = 3546) are presented in Table S5. Abbreviations: CAB‐LA, long‐acting cabotegravir; DALY, disability‐adjusted life year; ICER, incremental cost‐effectiveness ratio; MSM, men who have sex with men; PrEP, pre‐exposure prophylaxis.

At the CADTH sponsor‐submitted price of $10,260/year, expanding PrEP coverage among MSM in Montréal, leveraging CAB‐LA, was not expected to be cost‐effective under any expansion scenario. Rather, all health‐improving scenarios had median projected ICERs ranging from $922,900/DALY averted to $2,771,100/DALY averted. (Figure 3 and Table 2). Findings are similar under calculations without discounting (Table S6). Lower potential prices for CAB‐LA similarly failed to achieve cost‐effectiveness, including at the lowest modelled price of annual parity with oral PrEP (see Table S7).

**Table 2:** Cost‐effectiveness of scenarios expanding PrEP coverage for MSM with CAB‐LA and exposure risk prioritization in Montreal, USD 2021

			Health improving simulations (*N* = 3546^b^)			All simulations (*N* = 4000)		
Coverage %	Year achieved	Switching %	5‐%ile	Median ICER	95‐%ile	5‐%ile	Median ICER	95‐%ile
15%	2027	0	*308,100*	**922,900**	*8,691,500*	*326,000*	**1,913,500**	*Dominated* ^a^
		15	*473,100*	**1,030,300**	*5,103,800*	*570,000*	**2,437,500**	*Dominated* ^a^
		30	*520,500*	**1,555,700**	*11,317,500*	*577,900*	**2,915,400**	*Dominated* ^a^
		50	*724,400*	**2,057,700**	*52,384,300*	*864,400*	**2,931,300**	*Dominated* ^a^
		100	*1,122,400*	**2,771,100**	*17,625,500*	*1,163,900*	**6,043,700**	*Dominated* ^a^
	2032	0	*268,300*	**1,054,800**	*6,726,800*	*363,400*	**2,307,800**	*Dominated* ^a^
		15	*440,400*	**1,056,800**	*24,693,700*	*506,500*	**527,000,000**	*Dominated* ^a^
		30	*530,200*	**1,706,600**	*14,418,600*	*709,300*	**10,700,000**	*Dominated* ^a^
		50	*678,900*	**1,823,300**	*8,707,500*	*759,200*	**512,000,000**	*Dominated* ^a^
		100	*1,245,700*	**2,674,600**	*57,699,600*	*1,320,000*	**Dominated** ^a^	*Dominated* ^a^
30%	2027	0	*722,500*	**1,468,200**	*5,527,100*	*728,800*	**1,471,400**	*5,657,200*
		15	*743,500*	**1,626,700**	*6,471,800*	*743,500*	**1,626,700**	*6,471,800*
		30	*834,800*	**1,739,100**	*4,441,500*	*834,800*	**1,739,100**	*4,441,500*
		50	*866,300*	**1,938,700**	*5,694,700*	*888,100*	**1,943,400**	*5,710,400*
		100	*980,100*	**2,100,100**	*7,841,800*	*980,100*	**2,100,100**	*7,841,800*
	2032	0	*787,400*	**1,685,100**	*19,739,300*	*798,800*	**1,748,400**	*134,100,000*
		15	*777,200*	**2,150,500**	*14,573,300*	*818,800*	**2,282,400**	*Dominated* ^a^
		30	*836,500*	**2,385,200**	*10,897,700*	*842,300*	**2,500,900**	*688,200,000*
		50	*961,300*	**2,244,500**	*16,279,600*	*965,400*	**2,342,900**	*Dominated* ^a^
		100	*1,222,300*	**2,634,100**	*36,303,500*	*1,231,700*	**2,939,100**	*7,841,800*
40%	2027	0	*806,500*	**1,557,700**	*3,452,900*	*806,500*	**1,557,700**	*3,452,900*
		15	*852,100*	**1,624,300**	*4,314,400*	*852,100*	**1,624,300**	*4,314,400*
		30	*879,300*	**1,713,800**	*4,165,600*	*879,300*	**1,713,800**	*4,165,600*
		50	*955,000*	**1,760,500**	*4,751,000*	*955,000*	**1,760,500**	*4,751,000*
		100	*1,055,000*	**2,003,700**	*4,330,200*	*1,055,000*	**2,003,700**	*4,330,200*
	2032	0	*831,700*	**1,854,600**	*6,292,600*	*867,200*	**1,864,600**	*6,335,200*
		15	*941,900*	**1,997,900**	*6,811,800*	*941,900*	**1,997,900**	*6,811,800*
		30	*942,500*	**2,029,900**	*7,602,000*	*942,500*	**2,029,900**	*7,602,000*
		50	*1,029,200*	**2,094,200**	*6,831,400*	*1,034,700*	**2,102,400**	*6,912,100*
		100	*1,049,300*	**2,654,700**	*10,389,300*	*1,053,300*	**2,683,500**	*12,488,900*
50%	2027	0	*910,200*	**1,747,300**	*4,412,100*	*910,200*	**1,747,300**	*4,412,100*
		15	*921,800*	**1,780,500**	*4,056,200*	*921,800*	**1,780,500**	*4,056,200*
		30	*981,600*	**1,852,900**	*4,011,200*	*981,600*	**1,852,900**	*4,011,200*
		50	*1,040,600*	**1,886,900**	*4,450,000*	*1,040,600*	**1,886,900**	*4,450,000*
		100	*1,066,000*	**1,999,400**	*4,376,100*	*1,066,000*	**1,999,400**	*4,376,100*
	2032	0	*990,800*	**2,102,200**	*7,293,700*	*990,800*	**2,102,200**	*7,293,700*
		15	*996,900*	**2,125,600**	*5,010,900*	*1,006,200*	**2,134,700**	*5,258,600*
		30	*1,038,900*	**2,078,000**	*5,188,000*	*1,038,900*	**2,078,000**	*5,188,000*
		50	*1,160,500*	**2,259,900**	*6,542,200*	*1,160,500*	**2,259,900**	*6,542,200*
		100	*1,175,700*	**2,536,500**	*6,563,600*	*1,175,700*	**2,536,500**	*6,563,600*

Abbreviations: CAB‐LA, long‐acting cabotegravir; DALY, disability‐adjusted life year; ICER, incremental cost‐effectiveness ratio; MSM, men who have sex with men; PrEP, pre‐exposure prophylaxis.

^a^ An ICER value of “Dominated” refers to replicates where the number of new HIV infections was greater in the PrEP expansion than baseline scenario and the expansion scenario costs were higher than the baseline scenario.

^b^ Excluded simulations by scenario are presented in Table S5.

Due to the stochastic nature and low HIV incidence of the Montréal model, scenarios with small increases in PrEP coverage had worse HIV outcomes than baseline in some replicates. This led to ICER uncertainty intervals that sometimes included replicate ICERs that were negative or dominated. Such ICERs do not share a common interpretation with the positive ones in our graphical displays or linear analyses, and have thus been excluded from those displayed in Figure 3. See Table S5 for counts of excluded replicates by scenario.

As in Atlanta, median ICERs for PrEP expansion scenarios in Montréal tended to be more cost‐effective when fewer oral PrEP users switched to CAB‐LA. However, unlike in Atlanta, it was typically more cost‐effective to achieve targets by 2027 than by 2032 (*p* < 0.001). A series of Wilcoxon rank‐sum tests confirmed that all single‐ and multiple‐category increases in switching had statistically higher ICERs (*p* < 0.05) (Table S8). The impact of overall PrEP coverage on cost‐effectiveness was less consistent, except that 15% coverage was generally more cost‐effective than other coverage levels (*p* < 0.001) among health‐increasing replicates, and 50% coverage was usually less cost‐effective than 40% (*p* < 0.05) (Table S9).

## 4 DISCUSSION

Our analysis extends previous modelling findings and indicates that large‐scale PrEP expansions with CAB‐LA have the potential to be cost‐effective in high‐incidence settings similar to Atlanta. The probability of cost‐effectiveness increased when retaining the majority of current daily oral PrEP users on that regimen. Reducing the effective price of CAB‐LA via manufacturer rebates or other means could further improve the cost‐effectiveness and even achieve cost savings. It should be noted that the settings assessed here are both high‐income, and that while Atlanta may be regarded as “high‐incidence” for North America, similar incidence would not necessarily be considered “high” in other regions. Investigations within other geographic and economic contexts may yield different findings.

Our approach of using recent average oral PrEP prices for daily use as the default cost in Atlanta is key, and contrasts with prior estimates [21]. Prescribing patterns in the United States have been progressively shifting TDF/FTC users towards generics, accounting for 28% of all oral PrEP prescriptions in the first quarter of 2021 and up to 42% by the end of the third quarter [20, 34]. However, it is unclear how far this shift towards generics will continue, given that branded TAF/FTC prescriptions were still growing in 2021, potentially limiting near‐term average price decreases.

Notably, CAB‐LA recently received an “A” rating from the US Preventative Services Task Force [45], implying that under the Affordable Care Act, many insurers were required to cover it without cost‐sharing to users by September 2024. The analysis herein is timely, as there may be extensive negotiations surrounding manufacturer rebates during the scale‐up of this coverage mandate.

Under current conditions, our analysis does not support the cost‐effectiveness of extensive CAB‐LA‐based expansion in the low HIV incidence (1 per 1000 person‐year) and generic oral PrEP‐dominated setting of Montréal. The sponsor price for CAB‐LA is more expensive annually than frontline HIV ART options hampers the likelihood of a cost‐effective rollout, and even at lower prices, there remains a large number needed to treat in order to prevent one HIV acquisition [23]. Moreover, an increased number of outpatient visits appreciably increase the cost of CAB‐LA provision relative to oral PrEP even absent pharmaceutical price differences.

While large expansions of coverage with CAB‐LA appear unlikely to be cost‐effective, whether highly focused strategies providing CAB‐LA to individuals at substantial risk of HIV acquisition who are also facing barriers to oral PrEP access, adherence or persistence could be cost‐effective should be investigated. For example, fewer days with pills taken and high discontinuation rates of oral PrEP in younger and economically disadvantaged MSM [46] could make them good candidates for CAB‐LA [23].

It should also be noted that, as any setting achieves important declines in new HIV acquisitions, as Montréal has, further incidence reductions with the same tools will tend to have diminishing economic returns. However, complete transmission interruption, if achieved, would be a discrete event that averted an arbitrarily large number of DALYs over the population's full future, and may, therefore, require a different approach to economic valuation.

This investigation differs importantly in approach and findings from previous estimates. Our estimated ICERs are more favourable than those of Neilan and colleagues, which, besides using a much lower oral‐PrEP price comparator, also covers a shorter time horizon (10 years), and considers the outcomes of dichotomized patient‐level medication alternatives in a national setting rather than the population outcomes of expansion scenario possibilities with differing PrEP mixtures in a specific setting [21]. Our estimated ICERs are less favourable than those of Brogan and colleagues, which use a longer (lifetime) time horizon, and compare the dichotomized individual outcomes from CAB‐LA initiation plus switching to the lowest‐priced TDF‐FTC to outcomes from the lowest‐priced TDF‐FTC initiation with switching to branded‐price TDF/TAF, attributing the costs and benefits of distinct medications to the same initiation pathway [22].

Our analysis has several limitations. First, our findings are extensively calibrated to specific settings and are not directly generalizable elsewhere. Second, the prescribing landscape of oral PrEP in North America is actively changing. Reductions (increases) in the average price for prescriptions of oral PrEP would worsen (improve) the cost‐effectiveness of CAB‐LA‐based coverage expansion. However, we find that even under a hypothetical context of all oral PrEP use being the lowest‐priced generics, CAB‐LA PrEP expansions can be cost‐effective under appropriate conditions. Similarly, while we account for numerous sources of uncertainty in epidemic parameters and a wide range of potential effective prices for CAB‐LA, we did not attempt to model possible variations in PrEP programmatic costs or HIV‐care costs. HIV‐care costs could also be affected in unexpected ways by advancements in HIV care over time.

Finally, our analyses take as premises various potential PrEP coverage levels without attempting to estimate the start‐up and outreach costs necessary to achieve those coverage levels. In this way, our ICER estimates have a downward bias. However, to our knowledge, methods to achieve increases in coverage of modelled magnitudes in our settings have not yet been clearly demonstrated, though existing work can give suggestive insights. In perhaps the most relevant published costing, a pilot PrEP patient navigation intervention recruiting 61 participants in South Florida was extrapolated to Atlanta and projected to cost $81.45 monthly per individual to increase initiations by 21% among included individuals [47]. While revealing, the navigation intervention was also projected to have a maximum reach of 39% of the eligible MSM population, leaving open the cost of attaining our highest coverage level, even if programme average costs scaled unchanged to their full potential reach.

## 5 CONCLUSIONS

Our analysis suggests that expanding PrEP coverage among MSM utilizing CAB‐LA can be cost‐effective in high‐incidence and high oral PrEP cost settings such as Atlanta, so long as expansion is principally accessed by individuals with high exposure to HIV, most current users of daily oral PrEP remain on their current regimen, and the CAB‐LA price is reduced. At a cost of ≤$500/dose, CAB‐LA‐based PrEP expansion has the potential to be cost‐saving even relative to the lowest‐priced oral PrEP generics. Our analysis does not support the cost‐effectiveness of extensive CAB‐LA‐based expansion in lower HIV incidence settings such as Montréal, even at substantially lowered prices. However, highly targeted strategies providing CAB‐LA to high‐exposure individuals have yet to be investigated in such settings and remain an area for further research.

## FUNDING

Funding for SES, JAH, KMM, MM, DJD, RVB, M‐CB and DTD was provided by the NIAID, grant number UM1 068617. Funding for CMD, RMM, YX and MM‐G was provided by the Canadian Foundation for AIDS Research and the Canadian Institutes of Health Research. MM‐G's research programme is funded by a Canada Research Chair (Tier 2) in Population Health Modeling. CMD is supported by a doctoral award from the Fonds de recherche du Québec—Santé (FRQS).

## AUTHOR CONTRIBUTIONS

Conception of the research question: SES, DTD, M‐CB and JAH. Model development, parameterization, coding and simulation: KMM, M‐CB, MM and DTD (Atlanta model); CMD, RMM, YX and MM‐G (Montréal model). Costing, statistical analysis, figure and table creation: JAH. Drafting of the manuscript: JAH. Critical input into draft manuscript: all authors. Read and approved the final manuscript: all authors.

## COMPETING INTERESTS

The authors declare that they have no competing interests.

## Supporting information



Figure S1: Schematic of a hypothetical baseline projection compared to a PrEP expansion scenario with a 30% coverage target achieved in 10 years (2032). Bar heights indicate PrEP coverage by type. Total height is total PrEP coverage in 2042. Grey indicates the baseline 2022 oral PrEP coverage proportion that persists to 2042. Blue indicates oral PrEP coverage projected to increase without CAB‐LA introduction. Red indicates CAB‐LA coverage whether from baseline oral PrEP users who switched to CAB‐LA (bottom), CAB‐LA users who would have been recruited to oral PrEP between 2022 and 2042 if CAB‐LA were not an option (middle), and CAB‐LA users who would not have used oral PrEP in baseline projections (top). (PrEP: Pre‐exposure prophylaxis).
**Table S1**: Mapping of modeled HIV/AIDS progression categories to disability weights.
**Table S2**: Comparative Per‐PrEP‐Recipient Programmatic Costs of PrEP Provision in Atlanta by PrEP Option, 2020 USD.
**Table S3**: Comparative Per‐PrEP‐Recipient Programmatic Costs of PrEP Provision in Montreal, Canada by PrEP Option, 2021 CAD.
**Table S4**: Estimates of Annual Incremental Healthcare Costs due to HIV Per Person Living With HIV in the United States.
**Table S5**: Estimates of Annual HIV‐Related Healthcare Costs (CAD 2021) Per MSM living with HIV in Montreal, Canada.
**Table S5**: Counts of Montreal simulations which stochastically worsened health.
**Table S6**: Cost‐Effectiveness of scenarios expanding PrEP coverage for MSM with CAB‐LA in Montreal, USD 2021, DALYs calculated without discounting.
**Table S7**: Cost‐Effectiveness by hypothetical annual CAB‐LA price of scenarios expanding PrEP coverage for MSM with CAB‐LA in Montreal, USD 2021.
**Table S8‐A**: P‐Values of Wilcoxin rank‐sum tests comparing distributions incremental cost‐effectiveness ratios from different switching percentages. Health increasing simulations only .
**Table S8‐B**: P‐Values of Wilcoxin rank‐sum tests comparing distributions incremental cost‐effectiveness ratios from different switching percentages. All Simulations.
**Table S9‐A**: P‐Values of Wilcoxin rank‐sum tests comparing distributions incremental cost‐effectiveness ratios from different PrEP coverage levels. Health increasing simulations only.
**Table S9‐B**: P‐Values of Wilcoxin rank‐sum tests comparing distributions incremental cost‐effectiveness ratios from different PrEP coverage levels. All simulations.

## Data Availability

The model results that support the findings of this study are available from the corresponding author upon reasonable request.
