# Supplementary material for: Cost‐effectiveness of leveraging long‐acting injectable cabotegravir to expand PrEP coverage among MSM in two contrasting North American cities
Source: J Int AIDS Soc. 2026 Jan 19;29(1):e70061. doi: 10.1002/jia2.70061 (PMC12813553; doi:10.1002/jia2.70061)
Supplement: Supplementary file 1 — Figure S1: Schematic of a hypothetical baseline projection compared to a PrEP expansion scenario with a 30% coverage target achieved in 10 years (2032). Bar heights indicate PrEP coverage by type. Total height is total PrEP coverage in 2042. Grey indicates the baseline 2022 oral PrEP coverage proportion that persists to 2042. Blue indicates oral PrEP coverage projected to increase without CAB‐LA introduction. Red indicates CAB‐LA coverage whether from baseline oral PrEP users who switched to CAB‐LA (bottom), CAB‐LA users who would have been recruited to oral PrEP between 2022 and 2042 if CAB‐LA were not an option (middle), and CAB‐LA users who would not have used oral PrEP in baseline projections (top). (PrEP: Pre‐exposure prophylaxis). Table S1: Mapping of modeled HIV/AIDS progression categories to disability weights. Table S2: Comparative Per‐PrEP‐Recipient Programmatic Costs of PrEP Provision in Atlanta by PrEP Option, 2020 USD. Table S3: Comparative Per‐PrEP‐Recipient Programmatic Costs of PrEP Provision in Montreal, Canada by PrEP Option, 2021 CAD. Table S4: Estimates of Annual Incremental Healthcare Costs due to HIV Per Person Living With HIV in the United States. Table S5: Estimates of Annual HIV‐Related Healthcare Costs (CAD 2021) Per MSM living with HIV in Montreal, Canada. Table S5: Counts of Montreal simulations which stochastically worsened health. Table S6: Cost‐Effectiveness of scenarios expanding PrEP coverage for MSM with CAB‐LA in Montreal, USD 2021, DALYs calculated without discounting. Table S7: Cost‐Effectiveness by hypothetical annual CAB‐LA price of scenarios expanding PrEP coverage for MSM with CAB‐LA in Montreal, USD 2021. Table S8‐A: P‐Values of Wilcoxin rank‐sum tests comparing distributions incremental cost‐effectiveness ratios from different switching percentages. Health increasing simulations only . Table S8‐B: P‐Values of Wilcoxin rank‐sum tests comparing distributions incremental cost‐effectiveness ratios from different switching perce [file JIA2-29-e70061-s001.docx]

**Supplementary Material**

**
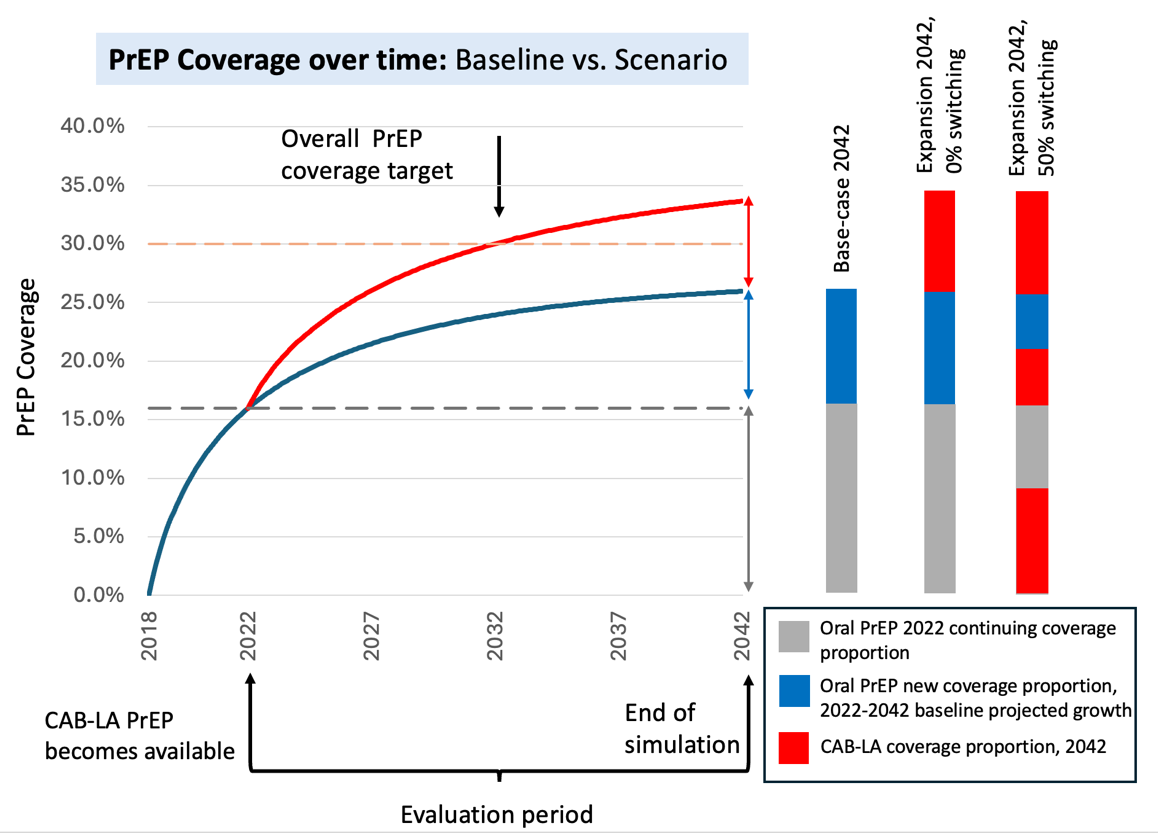
**

**Figure S1:** Schematic of a hypothetical baseline projection compared to a PrEP expansion scenario with a 30% coverage target achieved in 10 years (2032). Bar heights indicate PrEP coverage by type. Total height is total PrEP coverage in 2042. Grey indicates the baseline 2022 oral PrEP coverage proportion that persists to 2042. Blue indicates oral PrEP coverage projected to increase without CAB-LA introduction. Red indicates CAB-LA coverage whether from baseline oral PrEP users who switched to CAB-LA (bottom), CAB-LA users who would have been recruited to oral PrEP between 2022 and 2042 if CAB-LA were not an option (middle), and CAB-LA users who would not have used oral PrEP in baseline projections (top). (PrEP: Pre-exposure prophylaxis)

**Table S1: Mapping of modeled HIV/AIDS progression categories to disability weights**

| **Modeling Category** | **IHME GBD Category** | **Disability Weight** | **Source** |
| --- | --- | --- | --- |
| On ART | HIV/AIDS with antiretroviral treatment without anemia | 0.078 | IHME GBD 2019 [27] |
| CD4 Count 500+ | Early HIV without anemia | 0.012 | IHME GBD 2019 [27] |
| CD4 Count 350-500 | Early HIV without anemia | 0.012 | IHME GBD 2019 [27] |
| CD4 Count 200-350 | Symptomatic HIV without anemia | 0.274 | IHME GBD 2019 [27] |
| CD4 Count >200 | AIDS without anemia | 0.582 | IHME GBD 2019 [27] |

(IHME GBD: Institute for Health Metrics and Evaluation, Global Burden of Disease database)

**Table S2: Comparative Per-PrEP-Recipient Programmatic Costs of PrEP Provision in Atlanta by PrEP Option, 2020 USD**

| **PrEP Programmatic Process** | Unit Costs | Oral PrEP Interval (Wks) | CAB-LA Interval (Wks) | Yearly (52 wk) Cost: Oral PrEP | Yearly (52 wk) Cost: CAB-LA | Yearly Cost Difference |
| --- | --- | --- | --- | --- | --- | --- |
| Outpatient Visits | 104.00 | 12 | 8 | **451** | **676** | **225** |
| Injection Fee | 16.17 | N/A | 8 | **0** | **105** | **105** |
| HIV Testing^1^ | 9.09 | 12 | 8 | **39** | **59** | **20** |
| Non-HIV Lab Tests |  |  |  | **242** | **242** | **0** |
| Programmatic Total |  |  |  | **732** | **1082** | **350** |
| *Urinalysis (81005)* | *2.17* | *24* | *24* | *5* | *5* | - |
| *Urine GC/CT (87590 + 86631)* | *36.38* | *24* | *24* | *79* | *79* | - |
| *Rectal GC/CT (87590 + 86631)* | *36.38* | *24* | *24* | *79* | *79* | - |
| *Creatine (82565)* | *5.12* | *24* | *24* | *11* | *11* | - |
| *AST (84450)* | *5.18* | *24* | *24* | *11* | *11* | - |
| *ALT (84460)* | *5.30* | *24* | *24* | *11* | *11* | - |
| *Total Billirubin (82247)* | *5.02* | *24* | *24* | *11* | *11* | - |
| *Alkaline Phosphate (84075)* | *5.18* | *24* | *24* | *11* | *11* | - |
| *Syphilis (86592)* | *4.27* | *24* | *24* | *9* | *9* | - |
| *HCV (86803)* | *14.27* | *52* | *52* | *14* | *14* | - |

^1^ Assumes HIV IgG at CMS Reimbursement Rate

^2^ Assumes Liver tests (AST, ALT, total bilirubin, and alkaline phosphate), rectal chlamydia and gonorrhea tests, urine chlamydia and gonorrhea tests, syphilis tests, and creatine tests every 24 weeks, plus an annual HCV test, all at the RSSS Laboratory price

*(CAB-LA: long acting cabotegravir, CMS: Centers for Medicare & Medicaid Services, PrEP: pre-exposure prophylaxis, USD: United States Dollars)*

**Table S3: Comparative Per-PrEP-Recipient Programmatic Costs of PrEP Provision in Montréal, Canada by PrEP Option, 2021 CAD**

| **PrEP Programmatic Process** | Unit Costs | Oral PrEP Interval (Wks) | CAB-LA Interval (Wks) | Yearly (52 wk) Cost: Oral PrEP | Yearly (52 wk) Cost: CAB-LA | Yearly Cost Difference |
| --- | --- | --- | --- | --- | --- | --- |
| Outpatient Visits | 139.85 | 12 | 8 | **606.02** | **909.03** | **303.01** |
| Injection Fee | 14.90 | N/A | 8 | **0.00** | **96.85** | **96.85** |
| Pill Dispensing Fee | 9.00 | 12 | N/A | **39.00** | **0.00** | **-39.00** |
| HIV Testing^1^ | 7.74 | 12 | 8 | **33.54** | **50.31** | **16.77** |
| Non-HIV Lab Tests |  |  |  | **128.60** | **128.60** | **0.00** |
| Programmatic Total |  |  |  | **807.16** | **1184.79** | **377.63** |
| *Urinalysis* | *2.05* | *24* | *24* | *4* | *4* | - |
| *Urine GC/CT* | *13.27* | *24* | *24* | *29* | *29* | - |
| *Rectal GC/CT* | *13.27* | *24* | *24* | *29* | *29* | - |
| *Creatine* | *1.11* | *24* | *24* | *2* | *2* | - |
| *AST* | *1.11* | *24* | *24* | *2* | *2* | - |
| *ALT* | *1.11* | *24* | *24* | *2* | *2* | - |
| *Total Billirubin* | *1.11* | *24* | *24* | *2* | *2* | - |
| *Alkaline Phosphate* | *17.38* | *24* | *24* | *38* | *38* | - |
| *Syphilis* | *5.53* | *24* | *24* | *12* | *12* | - |
| *HCV* | *7.40* | *52* | *52* | *7* | *7* | - |

^1^ Assumes HIV IgG or Total Ac and Ag P24 at RSSS Laboratory price

^2^ Assumes Liver tests (AST, ALT, total bilirubin, and alkaline phosphate), rectal chlamydia and gonorrhea tests, urine chlamydia and gonorrhea tests, syphilis tests, and creatine tests every 24 weeks, plus an annual HCV test, all at the RSSS Laboratory price

^3^CAB-LA has no RAMQ standard price within Quebec, so the sponsor-submitted price to CADTH is assumed.

*(CAB-LA: long acting cabotegravir, CAD: Canadian Dollars, PrEP: pre-exposure prophylaxis, RSSS: Réseau de santé et des services sociaux)*

**Table S4: Estimates of Annual Incremental Healthcare Costs due to HIV Per Person Living With HIV in the United States**

| **Cost Category** | **People Living with HIV Annual Average Cost** | **People Living without HIV Annual Average Cost** | **Incremental Difference attributed to HIV Care** |
| --- | --- | --- | --- |
| **Outpatient Services** | $6,338 | $3,123 | **$3,215** |
| **Emergency Dept. Care** | $548 | $251 | **$297** |
| **Inpatient Care** | $4,032 | $1,380 | **$2,652** |
| **Pharmacy Utilization, including ART** | $29,434 | $1,344 | **$28,090** |
| **TOTAL** | **$40,352** | **$6,098** | **$34,254** |

*Adapted from Cohen et. al. 2020 [35]*

**Table S5: Estimates of Annual HIV-Related Healthcare Costs (CAD 2021) Per MSM living with HIV in Montréal, Canada**

| **Annual Cost, Treatment of HIV in Quebec** | **Total** |
| --- | --- |
| First Visit | $841 |
| Outpatient Follow Up in First Year | $531 |
| Outpatient Services in Subsequent Years | $629 |
| Emergency Dept. Care | $178 |
| Inpatient Care | $1,488 |
| ART Medications, Inexpensive | $6,208 |
| ART Medications, Expensive | $19,291 |
| Psychologist | $43 |
| Social Worker | $16 |
| **Average Annual Cost, Inexpensive ART** | **$9,304** |
| **Average Annual Cost, Expensive ART** | **$22,387** |

(ART: antiretroviral therapy, CAD: Canadian Dollars)

*Adapted from Ouellet et. al. 2015 [35]*

**Table S5: Counts of Montréal simulations which stochastically worsened health**

|  |  |  | Health harming simulations | | |
| --- | --- | --- | --- | --- | --- |
| Coverage % | Year Achieved | Switching % | More infections than baseline | More DALYs incurred than baseline | Total simulations omitted from primary analysis |
| 15% | 2027 | 0 | 21 | 30 | 34 |
|  |  | 15 | 23 | 38 | 40 |
|  |  | 30 | 16 | 29 | 31 |
|  |  | 50 | 20 | 28 | 34 |
|  |  | 100 | 24 | 33 | 39 |
|  | 2032 | 0 | 25 | 36 | 40 |
|  |  | 15 | 35 | 50 | 51 |
|  |  | 30 | 28 | 45 | 48 |
|  |  | 50 | 36 | 50 | 51 |
|  |  | 100 | 32 | 51 | 53 |
| 30% | 2027 | 0 | -- | 1 | 1 |
|  |  | 15 | -- | -- | -- |
|  |  | 30 | -- | -- | -- |
|  |  | 50 | -- | 1 | 1 |
|  |  | 100 | -- | -- | -- |
|  | 2032 | 0 | -- | 4 | 4 |
|  |  | 15 | -- | 6 | 6 |
|  |  | 30 | -- | 5 | 5 |
|  |  | 50 | -- | 7 | 7 |
|  |  | 100 | 1 | 5 | 5 |
| 40% | 2027 | 0 | -- | -- | -- |
|  |  | 15 | -- | -- | -- |
|  |  | 30 | -- | -- | -- |
|  |  | 50 | -- | -- | -- |
|  |  | 100 | -- | -- | -- |
|  | 2032 | 0 | -- | 1 | 1 |
|  |  | 15 | -- | -- | -- |
|  |  | 30 | -- | -- | -- |
|  |  | 50 | -- | 1 | 1 |
|  |  | 100 | -- | 1 | 1 |
| 50% | 2027 | 0 | -- | -- | -- |
|  |  | 15 | -- | -- | -- |
|  |  | 30 | -- | -- | -- |
|  |  | 50 | -- | -- | -- |
|  |  | 100 | -- | -- | -- |
|  | 2032 | 0 | -- | -- | -- |
|  |  | 15 | -- | 1 | 1 |
|  |  | 30 | -- | -- | -- |
|  |  | 50 | -- | -- | -- |
|  |  | 100 | -- | -- | -- |

(DALY: disability-adjusted life year)

**Table S6: Cost-Effectiveness of scenarios expanding PrEP coverage for MSM with CAB-LA in Montreál, USD 2021, DALYs calculated without discounting**

|  |  |  | Health Improving Simulations (N = 3,546) | | | All Simulations (N = 4,000) | | |
| --- | --- | --- | --- | --- | --- | --- | --- | --- |
| Coverage % | Year Achieved | Switching % | 5-%ile | **Median ICER** | 95-%ile | 5-%ile | **Median ICER** | 95-%ile |
| 15% | 2027 | 0 | *378,265* | **1,336,640** | *33,861,756* | *549,186* | **4,167,674** | *Dominated** |
|  |  | 15 | *632,530* | **1,979,532** | *9,919,477* | *745,469* | **16,300,000** | *Dominated** |
|  |  | 30 | *808,265* | **2,613,894** | *48,750,768* | *923,112* | **6,077,260** | *Dominated** |
|  |  | 50 | *957,464* | **3,487,132** | *21,848,152* | *1,310,471* | **14,900,000** | *Dominated** |
|  |  | 100 | *1,469,248* | **4,885,490** | *21,621,916* | *1,954,672* | **Dominated*** | *Dominated** |
|  | 2032 | 0 | *428,920* | **1,459,097** | *14,475,571* | *520,887* | **8,332,522** | *Dominated** |
|  |  | 15 | *608,521* | **1,533,986** | *17,902,230* | *674,344* | **Dominated*** | *Dominated** |
|  |  | 30 | *1,035,853* | **2,757,087** | *115,500,000* | *1,062,227* | **568,000,000** | *Dominated** |
|  |  | 50 | *854,739* | **1,721,144** | *10,011,274* | *1,102,476* | **Dominated*** | *Dominated** |
|  |  | 100 | *1,471,624* | **4,580,905** | *24,756,548* | *2,541,922* | **Dominated*** | *Dominated** |
| 30% | 2027 | 0 | *1,310,259* | **3,013,174** | *8,767,460* | *1,334,866* | **3,080,007** | *33,948,905* |
|  |  | 15 | *1,143,038* | **2,934,977** | *28,051,914* | *1,164,576* | **2,947,550** | *55,200,330* |
|  |  | 30 | *1,391,797* | **3,167,704** | *13,117,073* | *1,393,924* | **3,176,406** | *13,408,110* |
|  |  | 50 | *1,423,664* | **3,964,499** | *16,617,036* | *1,487,163* | **4,105,927** | *22,006,046* |
|  |  | 100 | *1,726,106* | **4,532,249** | *36,406,188* | *1,738,998* | **4,593,389** | *106,900,000* |
|  | 2032 | 0 | *1,235,541* | **2,755,509** | *24,567,524* | *1,285,333* | **2,853,175** | *Dominated** |
|  |  | 15 | *1,227,896* | **3,591,899** | *16,715,202* | *1,236,231* | **3,880,212** | *Dominated** |
|  |  | 30 | *1,275,640* | **3,835,551** | *35,199,292* | *1,296,952* | **4,275,971** | *Dominated** |
|  |  | 50 | *1,510,807* | **3,730,873** | *64,776,344* | *1,520,654* | **4,483,323** | *Dominated** |
|  |  | 100 | *2,030,566* | **5,065,496** | *20,489,770* | *2,075,738* | **5,554,747** | *Dominated** |
| 40% | 2027 | 0 | *1,417,829* | **2,857,071** | *8,427,519* | *1,417,829* | **2,857,071** | *8,458,390* |
|  |  | 15 | *1,568,987* | **3,081,060** | *11,326,491* | *1,587,269* | **3,083,355** | *11,965,646* |
|  |  | 30 | *1,593,568* | **3,149,074** | *10,911,744* | *1,593,568* | **3,149,074** | *10,949,615* |
|  |  | 50 | *1,611,813* | **3,413,202** | *13,164,482* | *1,611,813* | **3,413,202** | *13,205,115* |
|  |  | 100 | *1,924,989* | **4,083,623** | *13,617,638* | *1,924,989* | **4,083,623** | *13,657,875* |
|  | 2032 | 0 | *1,339,916* | **3,114,276** | *19,949,056* | *1,419,465* | **3,161,514** | *20,284,188* |
|  |  | 15 | *1,605,584* | **3,613,227** | *20,622,632* | *1,608,994* | **3,622,581** | *27,620,358* |
|  |  | 30 | *1,533,924* | **3,631,086** | *12,778,179* | *1,606,344* | **3,645,228** | *40,663,403* |
|  |  | 50 | *1,658,201* | **3,847,039** | *20,163,266* | *1,661,029* | **3,903,478** | *26,284,176* |
|  |  | 100 | *1,782,712* | **4,924,142** | *33,329,574* | *1,803,968* | **5,007,743** | *50,209,200* |
| 50% | 2027 | 0 | *1,529,212* | **3,248,776** | *9,461,963* | *1,529,212* | **3,248,776** | *9,496,761* |
|  |  | 15 | *1,621,532* | **3,307,086** | *9,702,452* | *1,621,532* | **3,307,086** | *9,737,410* |
|  |  | 30 | *1,760,382* | **3,396,328** | *11,792,637* | *1,760,382* | **3,396,328** | *11,833,444* |
|  |  | 50 | *1,812,495* | **3,608,679** | *11,461,857* | *1,812,495* | **3,608,679** | *11,500,195* |
|  |  | 100 | *1,891,306* | **3,937,937** | *9,489,163* | *1,906,238* | **3,939,223** | *10,045,118* |
|  | 2032 | 0 | *1,630,200* | **3,532,640** | *14,862,028* | *1,630,200* | **3,532,640** | *14,916,976* |
|  |  | 15 | *1,773,574* | **3,929,564** | *12,835,063* | *1,791,914* | **3,936,292** | *13,040,693* |
|  |  | 30 | *1,708,469* | **3,757,878** | *14,167,898* | *1,708,469* | **3,757,878** | *14,217,639* |
|  |  | 50 | *1,949,097* | **3,899,367** | *24,928,216* | *1,949,097* | **3,899,367** | *25,009,669* |
|  |  | 100 | *2,051,514* | **4,818,648** | *16,449,853* | *2,051,514* | **4,818,648** | *16,499,652* |

*(CAB-LA: long acting cabotegravir, DALY: disability adjusted life year, ICER: incremental cost-effectiveness ratio, PrEP: pre-exposure prophylaxis)*

**Table S7: Cost-Effectiveness by hypothetical annual CAB-LA price of scenarios expanding PrEP coverage for MSM with CAB-LA in Montreál, USD 2021**

| Coverage % | Year Achieved | Switching % | TDF-FTC Price Equivalence (2,629 CAD) | 5,000 CAD Annually | 7,500 CAD Annually | 10,000 CAD Annually | Sponsor Price (10,260 CAD) | 12,500 Annually |
| --- | --- | --- | --- | --- | --- | --- | --- | --- |
| 15% | 2027 | 0 | *342,277* | 653,959 | *978,427* | *1,302,895* | **1,336,640** | *1,627,364* |
|  |  | 15 | *376,922* | 864,313 | *1,378,761* | *1,922,937* | **1,979,532** | *2,433,318* |
|  |  | 30 | *536,229* | 1,214,975 | *1,879,861* | *2,544,746* | **2,613,894** | *3,209,631* |
|  |  | 50 | *587,391* | 1,380,856 | *2,352,269* | *3,380,225* | **3,487,132** | *4,408,180* |
|  |  | 100 | *699,775* | 2,038,918 | *3,403,741* | *4,745,904* | **4,885,490** | *6,088,068* |
|  | 2032 | 0 | *349,904* | 660,063 | *1,061,620* | *1,421,654* | **1,459,097** | *1,781,688* |
|  |  | 15 | *282,407* | 699,990 | *1,111,833* | *1,494,397* | **1,533,986** | *1,875,056* |
|  |  | 30 | *506,596* | 1,162,064 | *1,918,546* | *2,682,605* | **2,757,087** | *3,439,633* |
|  |  | 50 | *284,198* | 712,882 | *1,218,010* | *1,673,748* | **1,721,144** | *2,129,485* |
|  |  | 100 | *701,275* | 1,975,595 | *3,213,861* | *4,452,126* | **4,580,905** | *5,720,901* |
| 30% | 2027 | 0 | *913,067* | 1,557,800 | *2,249,518* | *2,941,235* | **3,013,174** | *3,632,952* |
|  |  | 15 | *870,850* | 1,512,187 | *2,188,418* | *2,864,649* | **2,934,977** | *3,540,880* |
|  |  | 30 | *894,557* | 1,614,252 | *2,364,725* | *3,092,061* | **3,167,704** | *3,819,397* |
|  |  | 50 | *1,074,022* | 1,960,555 | *2,913,000* | *3,865,445* | **3,964,499** | *4,817,890* |
|  |  | 100 | *1,154,689* | 2,166,507 | *3,273,597* | *4,415,661* | **4,532,249** | *5,536,702* |
|  | 2032 | 0 | *825,606* | 1,424,604 | *2,054,639* | *2,689,485* | **2,755,509** | *3,324,331* |
|  |  | 15 | *1,024,447* | 1,826,132 | *2,666,988* | *3,504,770* | **3,591,899** | *4,342,552* |
|  |  | 30 | *1,095,168* | 1,972,254 | *2,857,851* | *3,743,448* | **3,835,551** | *4,629,046* |
|  |  | 50 | *968,882* | 1,813,594 | *2,719,694* | *3,635,518* | **3,730,873** | *4,553,564* |
|  |  | 100 | *1,160,033* | 2,394,233 | *3,663,844* | *4,933,456* | **5,065,496** | *6,203,067* |
| 40% | 2027 | 0 | *894,371* | 1,503,322 | *2,146,739* | *2,790,156* | **2,857,071** | *3,433,573* |
|  |  | 15 | *939,955* | 1,601,024 | *2,300,160* | *3,007,497* | **3,081,060** | *3,714,453* |
|  |  | 30 | *941,182* | 1,627,063 | *2,350,881* | *3,074,024* | **3,149,074** | *3,795,657* |
|  |  | 50 | *958,657* | 1,712,798 | *2,517,571* | *3,329,703* | **3,413,202** | *4,132,570* |
|  |  | 100 | *1,052,974* | 2,021,118 | *3,007,119* | *3,982,214* | **4,083,623** | *4,957,308* |
|  | 2032 | 0 | *972,131* | 1,635,844 | *2,338,521* | *3,041,198* | **3,114,276** | *3,743,875* |
|  |  | 15 | *1,102,456* | 1,882,569 | *2,705,125* | *3,527,681* | **3,613,227** | *4,350,238* |
|  |  | 30 | *1,071,089* | 1,855,452 | *2,697,056* | *3,543,863* | **3,631,086** | *4,382,547* |
|  |  | 50 | *1,053,633* | 1,893,108 | *2,821,782* | *3,750,456* | **3,847,039** | *4,679,131* |
|  |  | 100 | *1,239,413* | 2,398,995 | *3,586,696* | *4,796,033* | **4,924,142** | *6,027,847* |
| 50% | 2027 | 0 | *1,034,284* | 1,723,042 | *2,448,201* | *3,173,359* | **3,248,776** | *3,898,518* |
|  |  | 15 | *1,027,979* | 1,734,850 | *2,482,111* | *3,229,371* | **3,307,086** | *3,976,631* |
|  |  | 30 | *1,028,640* | 1,761,243 | *2,538,374* | *3,315,506* | **3,396,328** | *4,090,410* |
|  |  | 50 | *1,070,827* | 1,864,219 | *2,691,918* | *3,521,142* | **3,608,679** | *4,362,845* |
|  |  | 100 | *1,116,994* | 1,986,208 | *2,925,672* | *3,842,579* | **3,937,937** | *4,759,486* |
|  | 2032 | 0 | *1,115,669* | 1,870,030 | *2,660,784* | *3,450,509* | **3,532,640** | *4,240,234* |
|  |  | 15 | *1,208,991* | 2,054,290 | *2,945,580* | *3,836,870* | **3,929,564** | *4,728,160* |
|  |  | 30 | *1,150,371* | 1,956,712 | *2,812,780* | *3,668,847* | **3,757,878** | *4,524,914* |
|  |  | 50 | *1,150,130* | 1,988,204 | *2,896,551* | *3,804,898* | **3,899,367** | *4,713,245* |
|  |  | 100 | *1,268,211* | 2,378,770 | *3,552,504* | *4,699,932* | **4,818,648** | *5,841,429* |

*(CAB-LA: long acting cabotegravir, CAD: Canadian dollars, DALY: disability adjusted life year, ICER: incremental cost-effectiveness ratio, PrEP: pre-exposure prophylaxis, TDF-FTC: tenofovir disoproxil fumarate/emtricitabine)*

**Table S8-A: P-Values of Wilcoxin rank-sum tests comparing distributions incremental cost-effectiveness ratios from different switching percentages. *Health increasing simulations only***

| **Switching Percent** | **0** | **15** | **30** | **50** | **100** |
| --- | --- | --- | --- | --- | --- |
| **0** | -- | 0.046 | 0.000 | 0.000 | 0.000 |
| **15** |  | -- | 0.006 | 0.000 | 0.000 |
| **30** |  |  | -- | 0.046 | 0.000 |
| **50** |  |  |  | -- | 0.000 |
| **100** |  |  |  |  | -- |

**Table S8-B: P-Values of Wilcoxin rank-sum tests comparing distributions incremental cost-effectiveness ratios from different switching percentages. *All Simulations***

| **Switching Percent** | **0** | **15** | **30** | **50** | **100** |
| --- | --- | --- | --- | --- | --- |
| **0** | -- | 0.009 | 0.000 | 0.000 | 0.000 |
| **15** |  | -- | 0.099 | 0.000 | 0.000 |
| **30** |  |  | -- | 0.033 | 0.000 |
| **50** |  |  |  | -- | 0.000 |
| **100** |  |  |  |  | -- |

**Table S9-A: P-Values of Wilcoxin rank-sum tests comparing distributions incremental cost-effectiveness ratios from different PrEP coverage levels. *Health increasing simulations only***

| **Coverage Percent** | **15** | **30** | **40** | **50** |
| --- | --- | --- | --- | --- |
| **15** | -- | 0.000 | 0.000 | 0.000 |
| **30** |  | -- | 0.633 | 0.151 |
| **40** |  |  | -- | 0.029 |
| **50** |  |  |  | -- |

**Table S9-B: P-Values of Wilcoxin rank-sum tests comparing distributions incremental cost-effectiveness ratios from different PrEP coverage levels. *All simulations.***

| **Coverage Percent** | **15** | **30** | **40** | **50** |
| --- | --- | --- | --- | --- |
| **15** | -- | 0.000 | 0.000 | 0.000 |
| **30** |  | -- | 0.141 | 0.749 |
| **40** |  |  | -- | 0.036 |
| **50** |  |  |  | -- |
